# How history and geography may explain the distribution in the Comorian archipelago of a novel mutation in DNA repair-deficient xeroderma pigmentosum patients

**DOI:** 10.1590/1678-4685-GMB-2019-0046

**Published:** 2019-12-13

**Authors:** Alain Sarasin, Patrick Munier, François Cartault

**Affiliations:** 1 Laboratory of Genetic Stability and Oncogenesis, UMR8200 CNRS, Gustave Roussy Institute, University Paris-Saclay, Villejuif, France.; 2 Department of Medical Genetics, INSERM U781 CHU Félix Guyon, La Réunion, France.

**Keywords:** DNA repair deficient diseases, sun-sensitivity, Comorian Archipelago, Bantu haplogroups, *XPC* gene

## Abstract

Xeroderma pigmentosum (XP) is a rare, genetic, autosomal nucleotide excision repair-deficient disease characterized by sun-sensitivity and early appearance of skin and ocular tumors. Thirty-two black-skinned XP from Comoros, located in the Indian Ocean, were counted, rendering this area the highest world prevalence of XP. These patients exhibited a new homozygous *XPC* mutation at the 3’-end of the intron12 (IVS 12-1G>C) leading to the absence of XPC protein. This mutation, characteristic of the consanguineous Comorian families, is associated with a founder effect with an estimated age of about 800 years. Analysis of mt-DNA and Y-chromosome identified the haplogroups of patients, who are derived from the Bantu people. Although the four Comorian islands were populated by the same individuals during the 7-10^th^ centuries, XP was found now only in the Comorian island of Anjouan. To avoid the slavery process caused by the arrival of the Arabs around the 11-13^th^ centuries, inhabitants of Anjouan, including XP-heterozygotes, hid inland of the island protected by volcanoes. This population lived with an endogamic style, without connection with the other islands. XP patients still live in the same isolated villages as their ancestries. Local history and geography may, thus, explain the high incidence of XP located exclusively in one island.

## Introduction

Xeroderma pigmentosum (XP) is a rare genetic autosomal recessive disease caused by a defect in the Nucleotide Excision Repair (NER) pathway (Kraemer and Sarasin, 2018; Sarasin *et al.*, 2019). This means that XP patients are unable to remove bulky DNA lesions induced in their genome following UV irradiation, cigarette smoking, or some other DNA-damaging treatments, such as anti-tumoral drugs (Saffi *et al.*, 2010). Biallelic mutations in one of the seven NER genes (*XPA* to *XPG*) are responsible for the DNA repair defect causing UV hypersensitivity and a very high increase in skin cancer incidence on exposed body sites (skin and eyes). About 50% of XP patients in the world are mutated in the *XPC* gene. The cells of these particular patients are deficient in global genome repair, but proficient in transcription-coupled repair (Sarasin and Stary, 2007; Hanawalt and Spivak, 2008). These patients are very sensitive to sun exposure, at a very high risk of developing skin cancers (basal-and squamous-cell carcinomas, and melanomas), but they show normal development and growth, and no neurological abnormalities.

We described a high incidence of black-skinned XP-C patients in the Comorian Archipelago with a frequency 100-200 times higher than in Europe (Kleijer *et al.*, 2008). These patients exhibited a novel homozygous splicing mutation in the intron 12 of the *XPC* gene leading to the absence of the wild type protein (Cartault *et al.*, 2011). This mutation had never been described before and we considered, at that time, that it was limited to the Comorian islands.

We report, here, that the “Comorian mutation” was in fact originated from the Bantu population living in East Africa, who peopled the Comoros during the 7^th^-10^th^ centuries. Heterozygous individuals probably populated the four Comorian islands similarly and simultaneously, but we explain now why the frequency of XP-C patients is extraordinarily high only in one of the four Comorian islands: The island of Anjouan (Nzwani in Comorian).

## Materials and Methods

### Subjects

The XP patients as well as some of their relatives were interviewed and examined by clinicians and geneticists at the Mayotte hospital and La Réunion University hospital as already reported (Cartault *et al.*, 2011). Skin biopsies to produce diploid skin fibroblasts and/or blood samples to isolate germline DNA were performed in agreement with the patients and their parents. All participants provided written informed consent and the study was done according to the guidelines of the Declaration of Helsinki. The genetic analyses were approved by the French Agency of Biomedicine (Paris, France) (N°2001/904 and AG08-0321 GEN of 27/09/2008) and approved by the European Commission “Geneskin: Genetics of Human Genodermatosis” (Brussels, Belgium, 2008). Patient photographs are shown with the authorization of the patients and parents.

### Molecular analysis of the cells from XP-C patients

The methods for analyzing the DNA repair deficiency, by measuring Unscheduled DNA Synthesis (UDS) on cultured skin fibroblasts and to confirm the clinical diagnosis of XP, have been previously published (Arnaudeau-Bégard *et al.*, 2003). Western blots and sequence analysis on proteins and DNA isolated from skin fibroblasts were carried out as described in Cartault *et al.* (2011).

### Determination of haplogroups of the XP-C patients

All haplogroups were performed by standard PCR with markers of HSV1 and HSV2 areas of mitochondrial DNA. The primers for DNA-Y analysis were selected from the ISOGG website (https://isogg.org/tree/index.html). The set was amplified and sequenced on a 3130 XL Applied Biosystem sequencer. The analysis of HVS1 and HVS2 variations for mitochondrial DNA was done using the Mitotools online analysis tool (http://www.mitotool.org/dloopRSRS.html). Analysis of markers of all known human haplogroups was previously done and showed that only haplogroups E, J and R were present in the sampling of Comorian patients. The in-depth analysis of subgroups by sequencing confirmed mainly their origin from West-Central Africa and their link with the Bantu people.

## Results

### The Archipelago of Comoros

**Figura 1:**
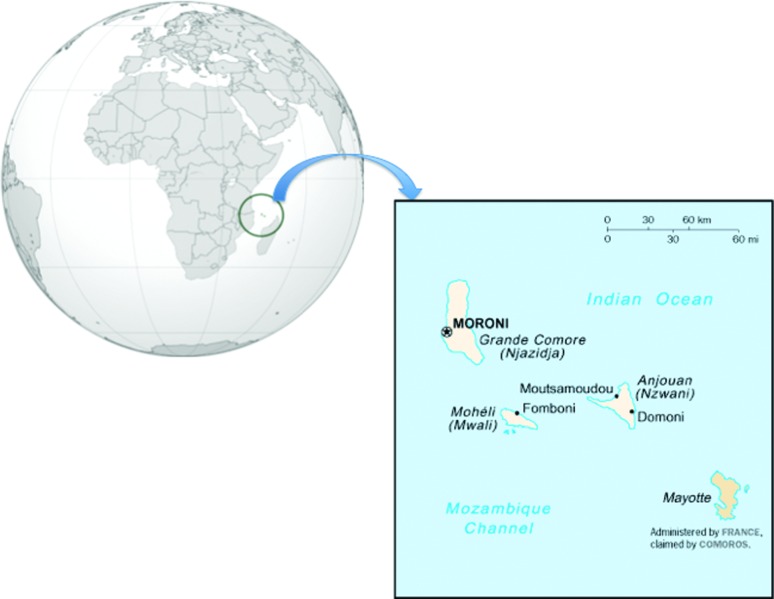
Location of the Comorian Archipelago between east Africa and Madagascar in the Indian Ocean. Four islands compose the Comorian archipelago. Three belong to the Federal Islamic Republic of the Comoros and the fourth one, Mayotte, is French (Modified from https://upload.wikimedia.org/wikipedia/commons/e/e3/Cn-map.png).

The Comoros archipelago is located in the western Indian Ocean, midway between the island of Madagascar and the coast of East Africa at the northern end of the Mozambique Channel. The archipelago is composed of four main islands (Figure 1). These islands represented a potential maritime crossroads between Bantu East Africa, Middle East, the Red See, the Arabic Peninsula and south-east Asian countries. In the past, the Indian Ocean was viewed as a closed, round sea, meaning there was much more sea traffic on this ocean compared to the Atlantic, for example. The name “Comoros” seems to derive from the Arabic “Kmr”, meaning “light in the sky” (Allibert, 2001), probably in reference to “the Cloud of Magellan” star constellation, whose position in the southern sky was used by seafarers as a means of finding the archipelago. The Comorian ethnic group is a synthesis of Bantu, Arab, Malay, and Malagasy cultures, and the main religion is Sunni Islam.

From the 7^th^ century, the Comoros were populated by Bantu Africans and the islands were used as a stepping stone for Middle Eastern traders and boats along the East African coast and for Austronesian traders travelling to Madagascar as well. The contacts between the Bantu population living on the coasts of East Africa and the Arabic traders coming from the Arabic Peninsula around the 10^th^ century lead to the emergence of the Swahili culture and language (Nurse and Philippson, 2003). The structure of the Swahili language was derived from the Bantu language but comported 40% of Arab words. Indeed, the Arab word “Swahil” means coast. The success of the Swahili civilization during the 9^th^-12^th^ centuries was linked to its commerce with the countries around the Indian Ocean. In the 12^th^ century, the Shirazi, from the city of Shiraz, which is now located in Iran, arrived on the Comoros, traveling and trading along the East African coast as far as India and the Maldives. Islamization of the Swahili coast and Comoros occurred, and the first mosque was built in Anjouan 800 years before present. Today, the oldest mosques found in the Comoros are all located in Anjouan (Sima and Domoni, 11^th^-12^th^) (Wright, 1984). Later the European colonization by the French, English and Portuguese traders and armies took over the administration of these countries. In 1886, the Comoros were established as a French Protectorate.

Following a vote for independence in 1975, today, three islands (Njazidja: La Grande Comore; Mwali: Mohéli, and Ndzwani: Anjouan) compose the Federal Islamic Republic of the Comoros, and the fourth one (Mayotte) remains French since 1843 (Figure 1). The Republic of the Comoros is a poor country, and has been rated as 160^th^ among 188 countries for the Human Development Index (https://en.wikipedia.org/wiki/Comoros).

### Xeroderma pigmentosum patients in Comoros

**Figura 2:**
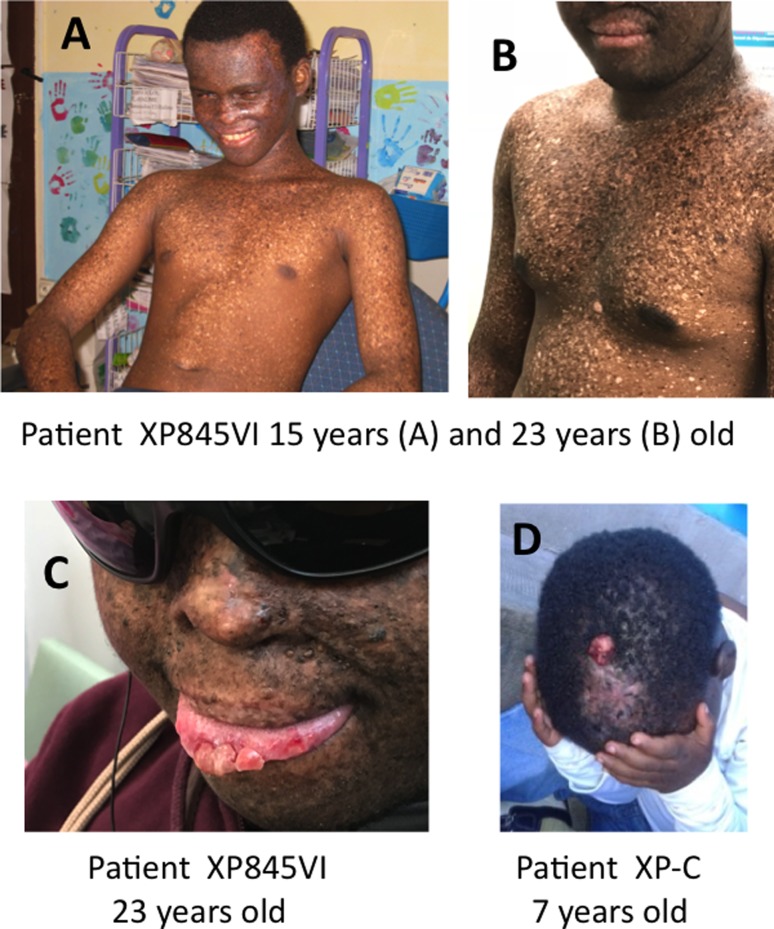
Representative photographs of XP-C patients from Comoros and skin tumors induced by sun-exposure. A) and B) Pictures of the same patient (XP845VI) at two different ages (15 and 23 years old) showing the increasing hypo- and hyper-pigmentation of the black skin. C) Shows a growing tumor on the tip of the tongue of the XP845VI patient at the age of 23 as well as small skin tumors on the face. D) Shows a squamous cell carcinoma on the head of a 7-year-old XP-C patient. (Pictures reproduced with the authorization of the patients and their parents).

**Figura 3:**
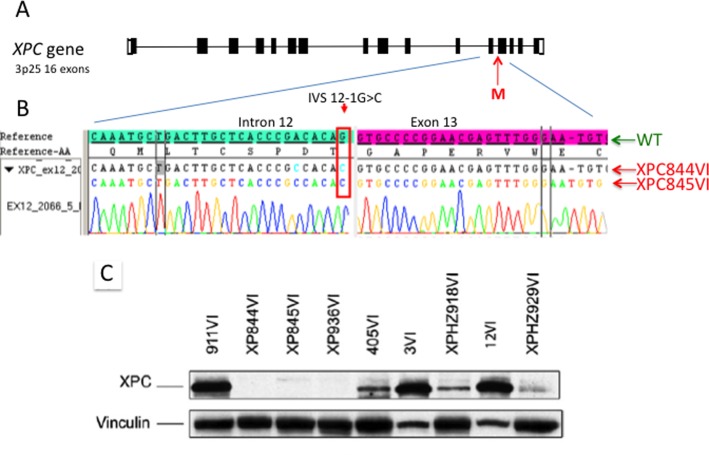
Point mutation in the *XPC* gene of all Comorian XP-C patients. A) The *XPC* gene is located on 3p25 and composed of 16 exons. B) The mutation (M), a G to C, is located on the splicing site on the last 3’ nucleotide of intron 12. The wild type sequence of intron 12 is indicated in green and two patient (XP844VI and XP845VI) sequences are given below. C) Represents a Western blot of the XPC protein in diploid skin fibroblasts. XP844VI, XP845VI and XP936VI correspond to three XP-C patients with no detectable XPC protein. XPHZ918VI and XPHZ929VI correspond to two heterozygous XP-C parents with about half of the normal amount of the XPC protein. The normal range of the XPC protein is given with the four other lanes corresponding to various wild type fibroblasts. Vinculin is shown as a marker of protein loading. (Adapted from Cartault *et al.*, 2011).

In the Comoros, about 32 black-skinned XP patients have been diagnosed in the last 15 years. Due to the high level of melanin, black XP patients are relatively more resistant to sun exposure than white-skinned patients. Sun exposure induced hypo- and hyper-pigmentation on black skin that progressed with age (compare Figure 2A and 2B). The first skin tumors they developed are in the eyes or on the tip of the tongue (Figure 2C). Later, they will develop skin tumors on exposed body sites (Figure 2D). In agreement with a normal development and normal neurology, the Comorian patients are all mutated on the *XPC* gene, exhibiting the same homozygous mutation (IVS 12-1 G>C) leading to an abnormal splicing site between intron 12 and exon 13 (Figure 3 A, B). Three abnormal mRNAs are produced, but none of them allowed the synthesis of a full-size XPC repair protein, as shown by western blot analysis (Figure 3C). These patients are, therefore, deficient in genome global excision repair, causing hypersensitivity to sunlight, showing numerous skin cancers, and ophthalmological anomalies, including early blindness. Because sun exposure is very high in Comoros, caused by the proximity with the equator and because of the absence of prevention and developed health care, these patients usually died in very dramatic conditions before the age of 15 (Cartault *et al.*, 2011).

**Figura 4:**
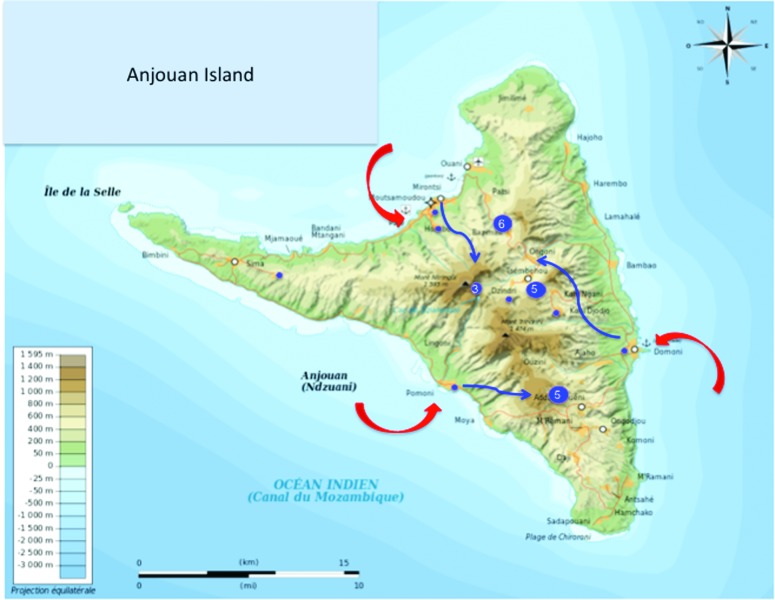
Localization of the villages where the XP-C families are still living in Anjouan. Anjouan is a small (424 km^2^) volcanic island. The red arrows indicate the major harbors where the Arabs probably arrived during the 10^th^ to 13^th^ centuries to colonize the island. The blue arrows suggest that the east African Bantu escaped the slavery process by moving to small villages with difficult access inside valleys between the volcanic mountains. The blue circles with numbers correspond to the number of XP-C patients found today in these villages. The small blue circles correspond to a unique XP-C patient found in each of the three major harbors and biggest cities of the island (Moutsamoudou, Pomoni, Domoni).

We were able to follow 18 of these patients in terms of both clinical and genetic analysis. Today, more than half of these patients live in Mayotte with free medical care provided by the French hospitals, but, in fact, all of them are originated only from the island of Anjouan. The familial structure in Comoros is “matrilocal”, meaning that after marriage the family lives in the village of the woman’s family. This situation is very well known to produce consanguinity and, therefore, the appearance of homozygous individuals. Moreover, the father or uncle is involved in the choice of the bride’s husband. This familial structure ensures a stable location of families over time because women are less subject to location change than men. Several generations, therefore, live at the same location due to this family structure. Indeed, most of the XP families are still now living in the middle of the volcanic Anjouan island in mountain villages with still very difficult access, far away from other cities and the seacoasts, meaning no genetic exchange with other individuals in the same island or from the other three islands. Interestingly, in some of these small villages we could find several XP patients, as much as four or six individuals in a small community, indicating that the number of heterozygotes should be elevated in the villages and the consanguinity factor rather high. In the three most developed cities on the coast, including the main town Moutsamoudou, only one XP family is found for each of these harbors, suggesting that the heterozygote individuals and consanguinity are essentially regrouped in closed villages, immune to genetic admixture (Figure 4).

### The genetics of the population of Comoros

The admixture analysis of the maternal and paternal contributions to the general Comoros population reveals the genetic pool to be essentially sub-Saharan African, distributed between 60% for males and 85% for females (Msaidie *et al.*, 2011). By computing genetic distances using haplogroups on the Y chromosome between the Comorian population and other source population, Ducourneau (2006) showed that the closest proximity is with sub-Saharan Africa and the Bantu expansion. This analysis also provides evidence for human Bantu migration from West-Central Africa down to the eastern/southern African coasts of the Indian Ocean (De Filippo *et al.*, 2012). With respect to male origins, the Arabic peninsula and Iran show a similar proximity (Msaidie *et al.*, 2011). The population dispersion was essentially done by sea routes, as shown by the lack of a Somalian population in Comoros. No detectable individual was coming from the Middle East with respect to the female contribution to Comorian peopling, in contrast to the Y chromosome contribution. This asymmetry suggests that an only men migration from the Middle East occurred, while women originated from East Africa stayed on the islands. This male expansion was linked to sea-borne commerce along the East African coast, as well as to the Islamic expansion. Some of these Arabic men married Comorian women of African and Swahili origins.

The distribution (Table 1) of the Y haplogroups in XP-C patients from Comoros, that we have established, [E1b1 (62%), J2a1 (15%), R1a1a (15%), E2b (8%)] is statistically different from the one published for the general population (*p*<0.01) (Msaidie *et al.*, 2011). Some relatively important haplogroups reported in the general Comorian population (E1b1a7 and E1b1a8a, 22% and 9% respectively) were completely absent from the XP-C population (Msaidie *et al.*, 2011). Although this difference was not statistically significant, probably due to the small number of XP-C patients, it is tempting to suggest that the fathers of the XP-C patients correspond to a small and restricted part of the African population originated from a limited number of heterozygote individuals peopling the Comoros in the past, at least based on the Y chromosome haplogroups. Indeed, African XP-C patients with the same mutation as the one found in Comorian patients, have been recently found in Kenya (data not shown), Mozambique, Zimbabwe, and north of South Africa (Coulombe *et al.*, 2016; Kgokolo *et al.*, 2019).

**Table 1:** Geographical origins of paternal (Y-chromosome) and maternal (mtDNA) lineages of the Comorian XP-C patients reported in this study.

Comorian XP-C patients	Y-chromosome		mtDNA	
DNA code	Haplogroups	Origins	Haplogroups	Origins
3003	E1b1a1a1f1	West-Central Africa	L1c	West-Central Africa
3538	E1b1a1a1f1	West-Central Africa	L0a2	South-East Africa
7961	E1b1a1a1f	West-Central Africa	L1c2a1	West-Central Africa
9199	E1b1a1a1f	West-Central Africa	L1c2a1	West-Central Africa
7053	E1b1a1a1f1a	West-Central Africa	L3e3	West Africa
2068	E1b1b1b	Nord-West Africa	L3d1a1a	West-Central Africa
2067	J2a1b1	East Africa	L3e3	West Africa
3124	J2a1b1	East Africa	L3e3	West Africa
1949	E2b	South-West-East Africa	L2a1	South Africa
7888	R1a1a1b1a2b1	Eurasia	L3f1b4a	East Africa Sahel Z.
9826	R1a1a1b1a2b1	Eurasia	L0a2	South-East Africa
2629	E1b1a1a1f	West-Central Africa	L3b	West Africa
AS970	E1b1a1a1f	West-Central Africa	L0d1b	Austral Africa

A similar analysis has been done by the Msaidie’s group on 577 mtDNA samples from Comorian islanders: 85% of the haplogroups (L0, L1, L2, L3) confirm that the ancestral population originated from south-eastern and eastern Africa corresponding to the Bantu expansion (De Filippo *et al.*, 2012), and 15% of Comorian mitochondrial origins are from southeastern Asia. This finding, based on a large Comorian population, is relatively close to the distribution that we found for the mtDNA of Comorian XP-C patients, except for the absence of a southeast Asian population in our patients (Table 1). Our XP-C maternal lineage corresponds to 100% (13/13 samples) of African origins (haplogroups L0, L1, L2 and L3) while the general Comorian population is around 85% of African origin (489/577 samples). This difference is not statistically significant probably due to the small number of XP-C patients. The mitochondrial haplogroups are more homogeneous in Comoros compared to the Y chromosome ones. This is in full agreement with the male-biased flow from Africa and Middle East to the Comoros, caused by male-dominated trade and religious propagation (Msaidie *et al.*, 2011).

Although the sample size of the XP-C patients is small, the XP families appear as a sample different from that of the total of Comorian inhabitants, particularly for the male lineage of XP parents. This result agrees well with the unique African/Bantu origin of the *XPC* mutation (for both lineages), which remained as such during centuries due to the absence of genetic exchanges with other populations, particularly Arabic and Asian ones.

### Why are all Comorian XP patients originated from only a single island: Anjouan?

When we tried to determine the geographical origins of the Comorian XP-C families, we discovered that all families were initially originated from a single island, Anjouan, and that no XP-C families were originated from any of the three other islands. Anjouan is a small volcanic island of 424 km^2^ with less than 300,000 inhabitants, and with no obvious difference from the other Comorian islands (Figure 4).

Like the other Comorian islands, Anjouan has been peopled by the east African Bantu between the 7^th^ and 10^th^ centuries. Since we know that the *XPC* founder mutation is present in East Africa, one can hypothesize that some individuals, men and/or women, who migrated from the mainland to the Comoros should have been asymptomatic XP heterozygotes.

Between the 11 and 13^th^ centuries, Arabs coming from the Arabic Peninsula and Shiraz (now Iran) followed the East African coast by boat for trade and eventually taking the black population as slaves. To avoid the slavery trade, it is possible that some of Anjouan inhabitants, including XP heterozygotes, may have fled to the central part of the island to hide in small valleys between the volcanic mountains. Due to the “matrilocal” familial structure and to the geography of the island, this people and their descendants stayed for centuries in the middle of the island, with almost no interaction with individuals living on the coast, coming from other islands or other countries (Blanchy, 2013). Although XP-C patients will not live very long due to their sun sensitivity in an equatorial country, their heterozygous parents had a normal lifespan because they were not sensitive to sun exposure and to cancer development. This hypothesis is strengthened by the fact that even today the families of XP patients live essentially inside the island in villages still difficult to access, and not along the coast where admixture and exchange with Arabic men would have been observed (see Figure 4). The populations living outside of cities and descending from Africans (the “Busmen” or *Wamatsaha*) were poor farmers and did not mix with the educated and sea traders from major towns of a Shiraz origin and an Islamic education (the *Makabaila*) (Blanchy, 2015). Even recently, peasants from African origin were obliged to move away from cities and coasts to places more and more difficult of access and farming, due to political reasons imposed by the rich traders of the three major harbors (Figure 4). This absence of mixing between different ethnic origins in the same island is a good picture of how the situation was centuries ago for the African individuals.

This hypothesis is in agreement with the age of the *XPC* mutations in Anjouan patients that we calculated to be around 800 years from now (Cartault *et al.*, 2011), which corresponds roughly to the arrival of the Arabs in Comoros. These 800 years correspond to the time the XP families and their descendants stayed together as a close bubble and without genetic exchange with other genetically different populations.

### Why such a story did not occur in the other Comorian islands is difficult to say?

May be the frequency of XP heterozygote Africans who crossed the sea from East Africa to Comoros, was low enough that statistically no or a too small number of such individuals arrived in the other islands? Indeed, we found only four different haplogroups in the Y chromosome and four different haplogroups in the mtDNA for the origins of the Comorian XP-C parents (Table 1), indicating that the number of ancestral XP heterozygotes was probably small during the Comoros peopling.

May be in the other islands, the Bantu Africans did not flee at the arrival of the Arabs, or did interact much more with commercial traders so that a high level of consanguinity did not occur as much as in Anjouan? It is interesting to note that the general population of Anjouan in the Middle Ages was known to be restricted to itself and to its own political situation (Blanchy, 2013). The international commercial trades were very active in Anjouan, but were conducted by a small minority of educated elite, living on the sea coasts, where the African farmers were excluded. The absence of mixing between poor farmers and educated individuals found in the classical Swahili society in Anjouan did not occur so much in the other Comorian islands, probably because of less competitive commercial activities (Blanchy, 2015).

Finally, the geography of the four islands is quite different, although all are volcanic islands. In Anjouan, there are several volcanoes with very isolated valleys of difficult access , where the XP families are still living now, in isolated small villages. In the three other islands, it appears to have been more difficult to hide and flee possible invaders. For example, in the biggest island (La Grande Comore), there is only one big volcanic mountain, and the rest of the island is flat. So, it is almost impossible to hide with families and children.

We can, therefore, hypothesize that the specific prevalence of XP-C patients in Anjouan could be due to a mixture of local historical and geographical circumstances leading to a specific evolution and life-style of the initial African peopling, including XP heterozygote individuals.

## DISCUSSION

In 2011 we reported a novel *XPC* mutation found only in Comorian islands (Cartault *et al.*, 2011)*.* It turns out that this mutation was probably coming from east/southeastern Africa because it has later been reported in the Kenyan, Mozambican, Zimbabwean and South African population (Kgokolo *et al.*, 2019). Heterozygous individuals for this mutation may have peopled the Comorian Archipelago between the 7^th^ to 10^th^ centuries. Probably, the four islands were accessed by these individuals, but only the Anjouan Island could accumulate, protect and propagate XP-C families. Several reasons could explain this geographical specificity: low number of heterozygous XP Africans crossing from the mainland; stronger protection against the Arab invasion of the Anjouan population which fled towards inaccessible small villages in the mountains; strong separation between poor farmers and the educated, rich, elite population of traders; lack of new genetic exchanges leading to strong consanguinity. This is confirmed by the haplogroups found in XP-C patients from Anjouan: all mothers were originated from Africa and more than 80% of the fathers were from the same area, in contrast to the male population of Comoros in general, which is more related to Arab countries. Although the *XPC* mutation probably arose randomly a long time ago in Africa, the same mutation, in the context of a close and consanguineous life style in Comoros, had been calculated to be as old as 800 years from now. This number corresponds fairly well to the time when the isolated communities in the middle of Anjouan were stably constituted in response to the Arab arrival. Hence, these data show how historical reports can be confirmed by analyzing rare genetic recessive human diseases.
